# Supplementary material for: The genome sequence of a picture-winged fly, Melieria picta (Meigen, 1826) (Diptera: Ulidiidae)
Source: Wellcome Open Res. 2025 Sep 15;10:515. [Version 1] doi: 10.12688/wellcomeopenres.24887.1 (PMC12553973; doi:10.12688/wellcomeopenres.24887.1)
Supplement: Peer review history

# Reviewer report: Reviewer response for version 1

**DOI:** 10.21956/wellcomeopenres.27414.r134654

**Published:** 2025-10-25

**Authors:** Teodora Staykova

**Affiliations:** 1 Plovdiv University, Plovdiv, Bulgaria

The genome of a picture-winged fly *Melieria picta* (Diptera: Ulidiidae) was studied based on a male specimen collected from Morston Creek, Blakeney Estuary, England, United Kingdom.  The methods used and statistical processing of the data are adequate to the purpose of the study. Results were obtained and are presented in 6 figures and 4 tables.Two haplotypes were identified. The haplotype 1  is scaffolded in 4 chromosomal pseudomolecules, of which 3 are autosomes and one X sex chromosome. No second sex chromosome was identified during the study, leading to the conclusion that male heterogamety of the XO type is observed in this species.

 The presented study is interesting and significant, as it fills in the missing information regarding the genome of this species. This study could serve as a basis for future population genetic studies within the species.

Are sufficient details of methods and materials provided to allow replication by others?

Yes

Is the rationale for creating the dataset(s) clearly described?

Yes

Are the datasets clearly presented in a useable and accessible format?

Yes

Are the protocols appropriate and is the work technically sound?

Yes

Reviewer Expertise:

Genetics, Population Genetics

I confirm that I have read this submission and believe that I have an appropriate level of expertise to confirm that it is of an acceptable scientific standard.

# Reviewer report: Reviewer response for version 1

**DOI:** 10.21956/wellcomeopenres.27414.r133375

**Published:** 2025-09-19

**Authors:** Mireille Steck

**Affiliations:** 1 University of Hawaiʻi at Mānoa, Honolulu, USA

This is a well-documented summary of a novel genomic assembly for a male *Melieria picta*. The methods used are appropriate and the statistics and summaries for the final genome are thorough. The quality of the genome and writing are both high, measures were taken during all steps of this experiment to generate a well curated final product. Documentation of all of these measures can be found in the protocols.io and githubs, which are well linked and easily accessible, with the exception of the link to "triaged" leading to the **Sanger Tree of Life Sample Preparation: Triage and Dissection**. The attachments in this protocol were not accessible at the time of this review for those without a protocols.io account. A similar protocol can be accessed through the Tree of Life Core Laboratory; however the results are significantly less detailed for decision making based on unique tissue types.

Are sufficient details of methods and materials provided to allow replication by others?

Yes

Is the rationale for creating the dataset(s) clearly described?

Yes

Are the datasets clearly presented in a useable and accessible format?

Yes

Are the protocols appropriate and is the work technically sound?

Yes

Reviewer Expertise:

Molecular biology, short read sequencing, biogeography and evolution

I confirm that I have read this submission and believe that I have an appropriate level of expertise to confirm that it is of an acceptable scientific standard.
